# Examining the relationship between endometriosis and psychological distress: roles of cultural background, body image and self-criticism in women's health

**DOI:** 10.1080/21642850.2025.2511980

**Published:** 2025-06-02

**Authors:** Shulamit Geller, Sigal Levy, Ronit Avitsur

**Affiliations:** a School of Behavioral Sciences, The Academic College of Tel Aviv-Yaffo, Tel-Aviv, Israel; b Statistical Education Unit, The Academic College of Tel Aviv-Yaffo, Tel-Aviv, Israel

**Keywords:** Endometriosis, body appreciation, self-criticism, cultural background, psychological distress

## Abstract

**Objective:**

Endometriosis, a chronic condition affecting 10-15% of women of reproductive age, often leads to psychological distress (PD), including depression and anxiety. This study examined how body image and self-criticism mediate the relationship between endometriosis and PD, comparing Israeli women to those from English-speaking countries, and explored the moderating role of cultural background.

**Methods:**

A cross-sectional survey was conducted with 437 women from Israel and English-speaking countries. Participants completed questionnaires to evaluate depression symptoms, anxiety, self-criticism, and body appreciation. Data were analyzed using multivariate MANOVA and moderated mediation models.

**Results:**

Women with endometriosis showed higher PD, with significant differences by cultural background. Israeli participants reported higher depression and anxiety. The mediation model showed that body image and self-criticism were significant mediators in the endometriosis-PD link, a process consistent across cultural groups. However, the direct effect of endometriosis on PD was stronger among Israeli women.

**Conclusion:**

Our findings highlight the impact of cultural context on PD on women with endometriosis, emphasizing body image and self-criticism as intervention targets. This study underscores the importance of culturally sensitive support for mental health in endometriosis, with implications for clinical practice and public health strategies.

## Introduction

Endometriosis is a chronic gynecological disease characterized by the growth of endometrial cells outside of the uterus, usually into the pelvis, diaphragm, and abdomen (Alimi et al., 2018). It is an unexplainable condition involving an uncertain and contested etiology (Painter et al., 2011) that affects approximately 10–15% of all women of reproductive age (Rowlands et al., 2021; Zondervan et al., 2020) across all ethnic and social groups (Bougie et al., 2022). Common symptoms are chronic pelvic pain, fatigue, congestive dysmenorrhea (heavy and painful menstruation), deep dyspareunia (pain associated with sexual intercourse), nausea, bowel and bladder issues (Becker et al., 2021), and infertility (Pope et al., 2015).

Endometriosis impacts multiple aspects of a woman’s life: emotional (Thomas et al., 2006), marital and sexual (Hudson et al., 2016; Sullivan-Myers et al., 2023), professional (Hansen et al., 2013), and psychological (for review see Donatti et al., 2017) among others. Maulenkul et al.’s (2024) recent integrative review of s systematic reviews found that women with endometriosis consistently experience higher levels of depression and anxiety compared to healthy women (in control groups), with prevalence rates ranging from 20% to 85%. These higher levels far exceed the prevalence seen in other chronic disease populations, including cancer (Brandenbarg et al., 2019), cardiovascular disease (Celano et al., 2016), and Type 1 diabetes (Hislop et al., 2008). High levels of anxiety and depression significantly are associated with symptom severity and overall health-related quality of life in endometriosis patients (Sullivan-Myers et al., 2021). Specifically, patients with high psychological distress (PD) tend to have a more negative sense of female identity, lower self-esteem, and poorer body image than those without PD (Facchin et al., 2017).

Despite growing interest in the link between endometriosis and PD, encompassing both depression and anxiety, research remains limited. There is a need for further studies that explore both direct and indirect effects, particularly the role of body image which has received little attention (Pehlivan et al., 2022; Volker & Mills, 2022). In particular, Facchin et al. (2017, 2018) showed that increased risk of depression and anxiety in women suffering from endometriosis may be attributed, in part, to changes in their quality of life due to impaired body image.

### Body image and body appreciation

Body image – a multifaceted and contextually influenced psychological experience involving both positive and negative perceptions and attitudes toward one’s body and appearance (Cash, 2004) – has gainedg gained growing interest among health researchers (Rodgers et al., 2023). Body image dissatisfaction is characterized by persistently negative cognitive and emotional perceptions of one’s body appearance and function (Cash, 2004). Positive body image, on the other hand, often referred to as body appreciation (Tylka & Wood-Barcalow, 2015), reflects respect and acceptance of one’s body and its functions alongside rejection of unrealistic body standards. Body appreciation refers to maintaining positive views of one's body, regardless of its physical appearance, and showing respect for it despite perceived imperfections or weight. It involves valuing the body's functionality and overall health (Tylka & Wood-Barcalow, 2015). It involves embracing one’s body despite its weight, shape, and imperfections and respecting it by attending to its needs and engaging in adaptive self-care behaviors (Avalos et al., 2005).

Studies have consistently linked body image issues with depression and anxiety in individuals with endometriosis (Facchin et al., 2017; Geller et al., 2021; Pehlivan et al., 2022). These issues include concerns about weight, size, general appearance, and body dissatisfaction (Facchin et al., 2017; Pehlivan et al., 2022). As shown in Tiggemann's (2011) work, internalization of appearance ideals and self-objectification are key mechanisms through which body dissatisfaction develops. This well-established relationship helps explain how the physical changes associated with endometriosis can negatively affect body image, contributing to greater psychological distress. Endometriosis- -related physical changes such as weight fluctuations due to hormonal treatments, scarring from surgeries, functional limitations caused by pain, and difficulties wearing desired clothing due to symptoms (Sayer-Jones & Sherman, 2023; Sullivan-Myers et al., 2021) may contribute to lower body appreciation (Geller et al., 2021) and have also been found associated with Psychological Distress (operationally defined here as symptoms of depression and anxiety) [PD] (operationally defined here as symptoms of depression and anxiety).

Evidence has further indicated that women with endometriosis are more concerned about their bodies than their healthy peers (healthy peers), often due to functional limitations and physical changes like pain, weight gain from hormonal therapy, surgical scars, and paleness from heavy bleeding and anemia (Van Niekerk et al., 2022; Volker & Mills, 2022). Symptoms of induced menopause, including fatigue, hot flashes, vaginal dryness, and reduced libido, also contribute to these concerns (Facchin et al., 2018). While poor body image may be directly linked to depression, a recent study demonstrated (Geller et al., 2021) that PD in women with endometriosis was mediated by lower body appreciation and increased self-criticism, though this finding was based on Israeli participants, thus limiting its broader applicability.

### Self-criticism

Self-criticism, marked by harsh self-judgment and punitive internal dialogue (Blatt & Zuroff, 1992), involves negative self-evaluation across traits, appearance, and performance (Shahar et al., 2015). It can mediate the relationship between body image and PD in women with endometriosis. As a maladaptive coping mechanism for perceived body flaws (Duarte et al., 2015), self-criticism is strongly linked to vulnerability to depression and anxiety, contributing to higher symptom severity, persistent self-devaluation, and lower quality of life especially in those with chronic illness (Pinto-Gouveia et al., 2014; Warren et al., 2016). Self-criticism significantly predicts PD across various chronic illnesses, including breast cancer (Campos et al., 2012), hypertension, heart failure, rheumatoid arthritis, hyperthyroidism (Pinto-Gouveia et al., 2014), and inflammatory bowel disease (Trindade et al., 2019). Recent studies have also linked self-criticism, body image, and PD in endometriosis, IBS, and fibromyalgia (Geller et al., 2021, 2024; Levy et al., 2024).

For women with endometriosis, negative self-perceptions often stem from deviations from societal body ideals. Internalizing societal messages about femininity, attractiveness, and reproductive capability may be associated with feelings of self-directed blame, unattractiveness or worthlessness (Gilbert, 2002; Quick, 2014; Tiggemann, 2011). These perceptions manifest as self-critical thoughts and emotions, such as guilt, shame, and anger (Gilbert & Procter, 2006), which can reinforce feelings of failure and a desire to escape and thus perpetuate a cycle of body shame and self-criticism (Geller et al., 2021). While some theoretical frameworks, like Gilbert's (2002), indicate a complex and cyclical relationship between body image and self-criticism, we ground our study on the Duarte et al.'s (2015) hypothesis that intense self-criticism related to body image mediates the link between chronic illness and symptomatology.

Addressing both body image appreciation and self-criticism could be vital for reducing PD, as interventions targeting these factors may disrupt cycles of negative self-evaluation and improve mental health outcomes for those with endometriosis.

### The present study

Historically, endometriosis research has focused on White middle-class women who delayed childbearing (Bougie et al., 2019), leaving a gap in our understanding of the experiences of women from a range of cultural and ethnic backgrounds. Despite its widespread prevalence and severe impact, much remains unknown about how endometriosis affects women outside this narrow demographic. The Global Study of Women’s Health (GSWH), conducted in 10 countries, revealed significant regional differences in quality of life among women with endometriosis (Nnoaham et al., 2011). Similarly, Chapron et al. (2016) found considerable variations in endometriosis symptoms and treatment across three diverse countries, emphasizing the need for more comprehensive and culturally diverse data to understand PD in endometriosis, including potential cultural and psychological influences.

In an attempt to bridge some of this above-described gap in the literature, the present study examined factors associated with PD in individuals with endometriosis, comparing two distinct cultural background groups: an Israeli sample and a sample from English-speaking countries, who typically serve as a baseline or control group in endometriosis research. This approach seeks to create a more inclusive and comprehensive model. Similar to most previous studies (e.g. Pehlivan et al., 2022), the present study focuses on a non – patient community sample, enhancing the generalizability of its findings.

Israel is a culturally and religiously diverse nation with traditional values (Geller et al., 2020a; Latzer et al., 2008). Its cultural emphasis on family, lower focus on physical appearance, and strong national identity have been linked to healthier body attitudes and self-directed behaviors (Zalcberg-Block et al., 2023). However, modernization and the adoption of Western values have begun to erode these protective factors (Heiman & Olenik-Shemesh, 2019). Additionally, as a familistic and pronatalist society, Israel has the highest fertility rates among OECD countries (OECD, 2019), which may intensify challenges for individuals with endometriosis, particularly due to infertility issues (Pope et al., 2015).

**Figure 1.**
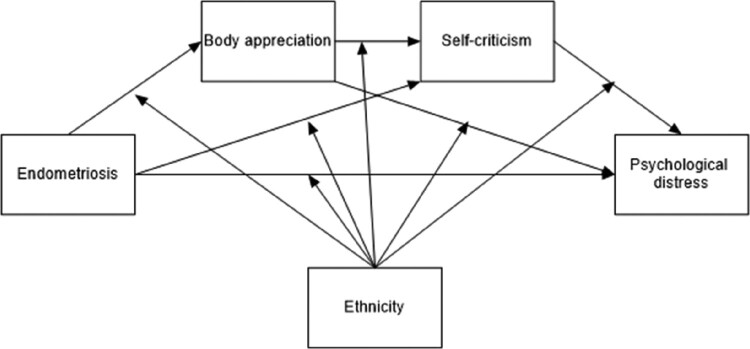
Hypothesized model.

Specifically, this study aimed to explore how body appreciation and self-criticism relate to Psychological Distress (operationally defined here as symptoms of depression and anxiety) in women living with endometriosis. Within this context, our study hypotheses were as follows: H1. Psychological distress, body appreciation, and self-criticism will differ by health status (women with endometriosis vs healthy peers), cultural background, and the interaction between these factors; H2a. body image appreciation followed by self-criticism will mediate the association between health status and psychological distress; and H2b. cultural background will moderate both the direct link between health status and psychological distress and the indirect link through body appreciation and self-criticism. Given the exploratory nature of this study, we aim to identify whether such differences exist, and what is the pattern of differences if they do. H2a and H2b are combined in the moderated mediation model described in Figure 1.

## Materials and methods

### Participants

The data for this study were obtained from a cross-sectional survey which was carried out both in Israel and in English-speaking countries (United Kingdom, New Zealand, Ireland, Canada, Australia) between 22-12-2020 and 15-12-2023. English speaking participants were chosen as the control group because many studies in this area have been conducted using this population (e.g. Cunnington et al., 2024). Participants were recruited via relevant online forums, including those specifically dedicated to supporting individuals coping with endometriosis and other general and international forums. Those who voluntarily consented to the study provided written informed consent and were then given a link to complete the 10–12 min survey electronically. Participants in both groups were informed that the goal of the study was to characterize psychological factors, such as depression, anxiety, and body image, in women coping with endometriosis. The Israeli women completed the questionnaires in Hebrew, while the English-speaking women completed them in English. The study group comprised women aged 20–50 who self-reported being diagnosed with endometriosis and lived either in Israel or an English-speaking country. The control group consisted of participants who reported no existing medical conditions. It should be noted that these diagnoses were not corroborated by medical records (healthy peers).

A total of 551 participants took part in the study. Of them, 53 participants who did not reside in Israel or an English-speaking country were excluded, as were 13 participants who were pregnant at the time of the survey and 48 who did not complete the survey. Our final sample therefore comprised 437 participants: 231 (53%) were from Israel (125 with endometriosis) and 206 (47%) were from English-speaking countries (153 with endometriosis). Of the English-speaking participants, 187 were from the United States, 185 from the United Kingdom, 123 from New Zealand, 82 from Ireland, 31 from Canada, and 9 from Australia. Descriptive statistics and a comparison of background measures for English-speaking and Israeli participants are presented in Table 1.

**Table 1.** Sample characteristics, group comparison, and Pearson correlations with the study outcomes.

Measure	Cultural background M(SD)/N(% of responders)		t(422)/χ^2^(1) for comparing cultures	Pearson correlations sample characteristics and outcome variables			
	Israel	English-speaking		Depression	Anxiety	BAS-2	SC
Age	31.2 (6.4)	33.8 (7.8)	3.8**	−0.01	−0.04	−0.00	−0.06
In a relationship Yes No	161 (70) 70 (30)	161 (79) 42 (21)	5.2*	0.00	0.01	−0.01	0.01
Has children Yes No	51 (24) 163 (76)	71 (54) 61 (46)	32.1**	−0.02	−0.01	−0.05	0.00

Note: Due to missing values, the sample size for these comparisons is smaller than for the main study model. * *p* < 0.05, ** *p* < 0.01. BAS-2 Body appreciation scale. SC – Self criticism

### Measures

#### Demographics

Participants reported their age, marital status, and number of children. In addition, they reported whether they were currently pregnant or suffered from other chronic illnesses (e.g. high blood pressure, diabetes, heart condition, asthma, irritable bowel disease). Women who reported being diagnosed with endometriosis also reported the time since their diagnosis, the time since first noticing endometriosis symptoms, and their age when first seeking medical help. They were asked to rate their endometriosis-related pain levels in the past month on a 7-point Likert-type scale ranging from 1 (no pain) to 7 (unbearable pain).

#### Depression

Depression was evaluated using the 9-item Patient Health Questionnaire (PHQ-9) (Geulayov et al., 2009; Kroenke et al., 2001 Hebrew translation). All items were rated on a 4-point scale ranging from 0 (not at all) to 3 (nearly every day). Total scores were obtained by summarizing the scores of all items. The total score ranges from 0 to 27 with higher scores indicating higher levels of depression. Internal consistency of the original PHQ-9 was .89_ and .86, in the translated and validated Hebrew measure was shown to be adequate (Cronbach α 0.77), while in the present study, McDonald’s ω was 0.88.

#### Anxiety

Anxiety was evaluatedusing the Generalized Anxiety Disorder Scale (GAD-7) (Löwe et al., 2008; Spitzer et al., 2006 Hebrew translation). The GAD-7 is a 7-item generalized anxiety measure (panic disorder, social anxiety disorder, and post-traumatic stress disorder). All items were rated on a 4-point scale ranging from 0 (not at all) to 3 (nearly every day). Total scores were obtained by summarizing the scores of all items. The total score ranges from 0 to 21 with higher scores indicating higher levels of anxiety. Internal consistency of both the original and the translated and validated Hebrew GAD-7 was high (Cronbach = .92). In this study McDonald’s ω was 0.93.

#### Self-criticism

Self-criticism was assessed using six items from the Depressive Experiences Questionnaire (DEQ; Blatt et al., 1976), a 66-item scale that measures predispositional patterns related to depressive states. The six-item measure used in this study (DEQ-SC6; Rudich et al., 2008) was identified and validated in previous research as an effective measure of self-criticism in Hebrew. This subscale reflects concerns about failure and the inability to meet high standards. All items were rated on a 7-point scale ranging from 1 (strongly disagree) to 7 (strongly agree). Scores were obtained by averaging across items, with higher scores indicating greater self-criticism. The Internal consistency of the original measure was adequate (Cronbach’s = .75), and in this study McDonald’s ω was 0.91.

#### Body appreciation

Body appreciation was measured using the Body Appreciation Scale-2 (BAS-2) (Geller et al., 2020; Tylka & Wood-Barcalow, 2015 Hebrew translation). This is a 10-item measure that assesses how people accept their body, respect and care for their body, and protect their body from unrealistic beauty standards. Each item ranges from 1 (never) to 5 (always). An overall BAS-2 score was computed as the mean of all items, with higher scores indicating greater body appreciation. The internal consistency of the original BAS-2 ranged from 0.89–0.95. The translated and validated Hebrew version showed adequate internal consistency (McDonald’s ω = .93), and in this study McDonald’s ω was also 0.93.

### Statistical analysis

Descriptive statistics are presented as M(SD) for continuous variables or N(%) for count data. A group comparison of the background variables was done using independent samples t-tests or the Chi-square test. Pearson correlations were used to estimate the relationship between variables. A multivariate MANOVA, followed by a univariate ANOVA, was used to test the joint effects of country of origin and health status on the primary outcome variables. Partial Eta squared (η_p_^2^) was used to estimate the effect size in both the univariate and multivariate analyses. Process model 92 and a custom-made model were used to test the hypothesized model.

### Ethical approval

The study was conducted in accordance with the principles of the Declaration of Helsinki, and ethics approval was obtained from the Academic College of Tel-Aviv Yaffo ethics committee (approval code: 2020017[2020], 186[2023]), prior to data collection.

## Results

**Figure 2.**
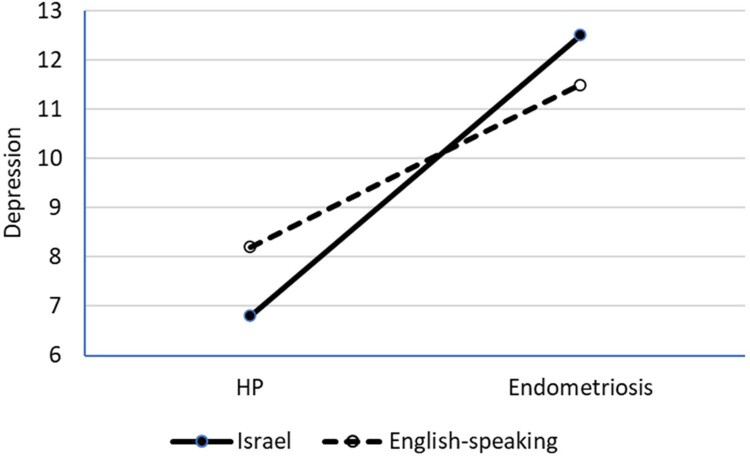
Cultural background by health condition interaction affecting depression.

**Figure 3.**
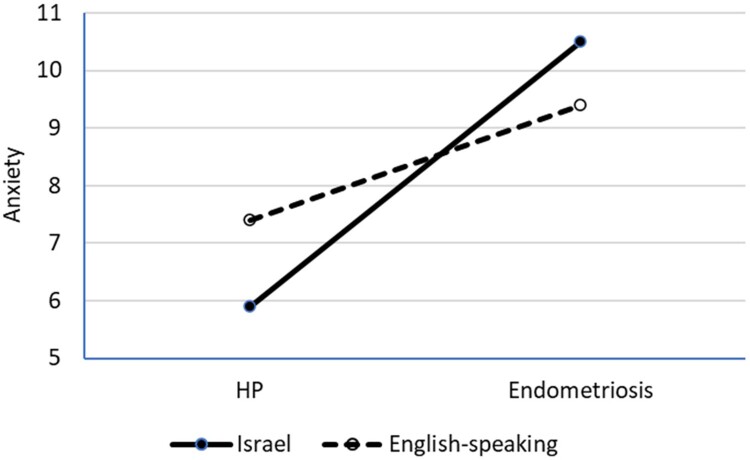
Cultural background by health condition interaction affecting anxiety.

A MANOVA model was used to explore the nature of the combined relationships between the country of origin, endometriosis, and the main study outcomes. As age, relationship status, and having children did not correlate with any outcome variables, no covariates were included in the model. This analysis revealed a significant main effect of country of origin (F(4, 430) = 7.6, *p* < 0.01, ᵑ_p_^2^ = 0.07) and health condition (F(4,430) = 14.1, *p* < 0.01, ᵑ_p_^2^ = 0.12) and a significant interaction between these factors (F(4, 430) = 2.6, *p* = 0.038, ᵑ_p_^2^ = 0.02). A follow-up univariate analysis showed a significant effect of health status on all outcomes, higher body appreciation among Israeli participants and significant interactions associated with depression and anxiety. The results are presented in Table 2 and the significant interactions are shown in Figures 2 and 3.

**Table 2.** Joint effect of health status and cultural background on the outcomes: means, standard deviations, test statistics and effect sizes.

	Israel		English-speaking				
	HP	Endo	HP	Endo	F(1, 433) value (ᵑ_p_^2^)		
	M (SD)	M (SD)	M(SD)	M(SD)	Cultural background	Health	Cultural background by health
Depression	6.8 (5.1)	12.5 (6.3)	8.2 (6.0)	11.5 (5.8)	0.2 (.00)	55.4** (.11)	4.1* (.01)
Anxiety	5.9 (4.9)	10.5 (6.0)	7.4 (4.9)	9.4 (5.4)	0.1 (.00)	34.9** (.08)	5.2* (.01)
Body Appreciation	3.7 (0.8)	3.4 (0.8)	3.3 (0.8)	3.0 (0.7)	23.6** (.05)	11.9** (.03)	0.0 (.00)
Self-criticism	4.4 (1.1)	4.7 (1.1)	4.3 (1.2)	4.8 (1.0)	0.1 (.00)	14.0** (.03)	0.7 (.00)

Note*:* HP = Healthy Peers, Endo = Endometriosis * *p* < 0.05, ** *p* < 0.01.

**Figure 4.**
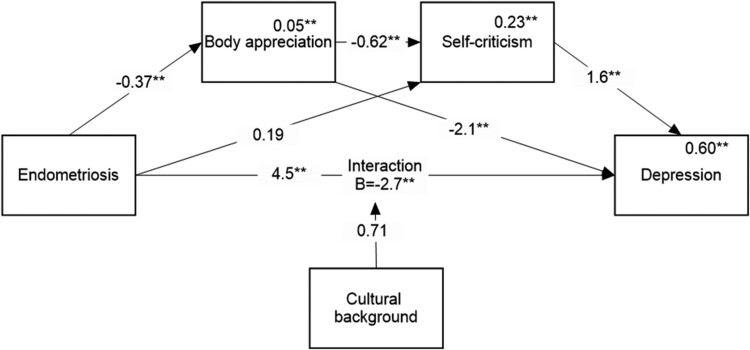
Mediation model for predicting depression. Note: Numbers above the lines are unstandardized regression coefficients. Numbers above the variables’ names are multiple squared correlations. **p < 0.01.

**Figure 5.**
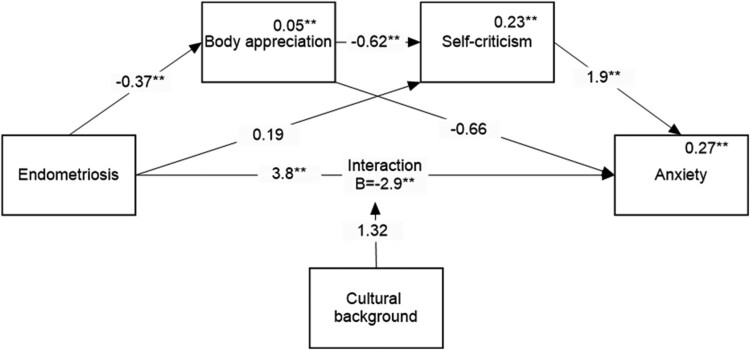
Mediation model for predicting anxiety. Note: Numbers above the lines are unstandardized regression coefficients. Numbers above the variables’ names are multiple squared correlations. **p < 0.01.

Regarding the moderation model, the hypothesized model yielded no significant moderated mediation; in other words, no significant differences were found between the countries of origin in any of the indirect paths. However, the direct effect was moderated by the country of origin, and we therefore revised the hypothesized model so that it only included moderation of the direct effect and repeated the analysis. The results of the revised analysis are presented in Figures 4 and 5.

We discovered two significant indirect paths: one through body appreciation (*B* = 0.79, 95% CI = [0.39, 1.3) and the other through body appreciation, linked to self-criticism and associated with depression (*B* = 0.37, 95% CI = [0.18, 0.59]). In addition, we found that the direct effect was moderated by country of origin, showing that endometriosis has a stronger adverse effect on depression among Israelis than among residents of English-speaking countries.

Similar results, presented in Figure 5, were obtained for anxiety. We discovered one significant indirect path going through body appreciation, linked to self-criticism and associated with depression (*B* = 0.42, 95% CI = [0.22, 0.67]). In addition, we found that the direct effect was moderated by country of origin, showing that endometriosis had an adverse effect on anxiety among Israelis but not among residents of English-speaking countries.

The simple effects for both models are shown in Table 3.

**Table 3.** Simple effects analysis of the moderated direct effects of endometriosis on PD. (Numbers are effect [95%CI]).

	Israel	English-speaking
Depression	4.5** [3.2, 5.9]	1.82* [0.24, 3.4]
Anxiety	3.8** [2.5, 5.1]	0.88 [−0.67, 2.4]

Note: * *p* < 0.05, ** *p* < 0.01.

## Discussion

The detrimental effects of endometriosis on PD have been well-established in earlier studies (Maulenkul et al., 2024; van Barneveld et al., 2022); however, cultural differences in these effects remain understudied. The present study aimed to enrich the existing body of knowledge on the role of cultural background in the relationship between endometriosis and PD by comparing its underlying mechanism among women in Israel and English-speaking countries.

The results of our study align with existing findings, showing that women with endometriosis experience higher levels of depression and anxiety than their healthy peers. Cultural background was also found to be important as PD symptoms were more pronounced among women with endometriosis residing in Israel than among those residing in English-speaking countries. The moderated mediation model used in our study helps explain this difference. As is typical in mediation models, the link between endometriosis and PD was analyzed through two components. The first component is the indirect effect, which outlines a sequence according to which endometriosis is associated with lower body appreciation higher self-criticism, and ultimately, higher PD. This indirect pathway did not significantly differ between cultural groups, suggesting that the underlying psychological process is consistent across cultures. The mediating roles of body image and self-criticism in the relationship between endometriosis and PD appear to be universally intertwined. Specifically, self-criticism is uniquely connected to body image as a maladaptive coping strategy, in which negative perceptions of the body intensify harsh self-judgment and internalized criticism (Duarte et al., 2015; Gilbert, 2002). This connection, fueled by societal pressures to meet certain physical ideals (Quick, 2014), appears to transcend cultural boundaries, thus explaining its consistency across diverse backgrounds (Geller et al., 2021; Gilbert & Procter, 2006). It can be further understood through the body dissatisfaction-driven hypothesis, which suggests that feelings of body-related inadequacy can extend to broader self-perception, fostering a self-deprecating cycle that lowers self-esteem – a key risk factor for depression (Pehlivan et al., 2022). This is particularly relevant for women of reproductive age, for whom body image is central to self-esteem (Sharpe et al., 2018).

The second component of the mediation model, the direct effect, revealed a significant difference, with endometriosis having a stronger impact on Israeli women than on women from English-speaking countries. Although this study did not examine the mechanisms underlying this effect, societal devaluation of stigmatized identities is a known contributor to self-criticism and shame (Luoma & Platt, 2015). It is possible that culturally specific pressures in Israeli society, where family and motherhood are highly valued (Zalcberg-Block et al., 2023), intensify the psychological toll of endometriosis. Infertility, defined as the inability to conceive after 12 months of unprotected intercourse, affects 30–50% of women with endometriosis (Vitale et al., 2017), and previous studies have shown a link between infertility and negative body image (Facchin et al., 2017). Moreover, women with endometriosis often perceive that infertile women are less valued – a belief that is associated with poorer mental health (Facchin et al., 2018). Research in pronatalist societies like Israel has suggested that women face greater psychological strain when dealing with infertility and that this strain is intensified by the societal emphasis on motherhood (Hashiloni-Dolev, 2018).

Taken together, these findings suggest that in Israel’s pronatalist context, infertility can exacerbate PD, which is often accompanied by self-criticism and a sense of defeat due to perceived deviation from the cultural ideals of womanhood and family (Birenbaum-Carmeli & Carmeli, in press). Moreover, as childbearing is highly valued and seen as a civic duty in Israel, childlessness carries significant stigma and social consequences (Yeshua-Katz, 2018), which can lead to isolation, social rejection, and worsened PD outcomes. In contrast, most English-speaking countries are more individualistic and less pronatalist, and this may reduce stigma and public pressure around endometriosis and infertility, alleviating stress for affected women. Less stigma may create a more accepting environment, thus normalizing the experience and facilitating help-seeking behaviors that mitigate the psychological burden associated with these conditions (Greil et al., 2011). However, it should be noted that individualistic cultures may impose different pressures, such as competitiveness and self-blame (Humphrey & Bliuc, 2021), though these were less prominent in the current study.

The findings of the present study have significant clinical implications. First, as perceptions of their illness may be associated with patients’ willingness to seek care and influence the quality of care they receive (Kocas et al., 2023), understanding sociocultural variations in infertility stigma is crucial for supporting mental health. Previous studies have indicated that women from pronatalist backgrounds and certain ethnic backgrounds may face heightened stigma, linked to increased PD (Denny et al., 2011). Clinicians should consider these cultural factors in their patient assessments and tailor counseling to address these specific challenges, thereby fostering resilience and enhancing well-being (Cunnington et al., 2024). Second, clinicians are advised to address body image and self-criticism concerns in individuals with endometriosis as this may help reduce related PD. Understanding the sources of self-criticism and shame (e.g. infertility stigma) encountered by women with endometriosis from diverse backgrounds can further enhance interventions at individual, community, and societal levels.

The present study contributes to the existing body of knowledge by validating previous findings across women from diverse cultural backgrounds and elucidating the distinct coping mechanisms they employ to manage endometriosis. Nevertheless, the study is not without limitations. Its foremost limitation is its cross-sectional design, which restricts the ability to draw causal inferences. The promising results obtained from path analysis support such conclusions and underscore the necessity for longitudinal studies that explore the influence of cultural backgrounds on coping strategies for chronic illnesses. A second limitation pertains to the validity of the model, which could be enhanced by accounting for variations in symptom severity and endometriosis type. Third, illness status was self-reported by participants, and their health status was not independently verified. Fourth, participants were recruited online, and previous research has indicated that the recruitment method can influence the responses of women with endometriosis. Specifically, women who responded online may have been more computer-savvy and inclined to use technology compared to those who would have answered written questionnaires. Additionally, women who engage in online forums or support groups may differ psychologically from those who do not. Fifth, treating Israel and English-speaking countries as homogeneous groups overlooks cultural diversity within them, leaving the nuances of each population unaddressed. Lastly, while this study speculates on the potential role of perceptions of infertility and childlessness, differences among women from various cultural backgrounds can also be attributed to other factors, such as varying access to diagnosis, treatment, and psychological support. Future research should examine specific cultural beliefs about femininity, body image, infertility, and stigma among women with endometriosis. While body functionality was outside the scope of the current research, delving into its role may provide a deeper wholistic understanding of body image that considers adaptive and resilience-building perspectives. We also recommend investigations of underlying mechanisms contributing to PD across different cultural groups to identify potential similarities or differences, thus emphasizing the need for more inclusive research.

In conclusion, the findings of the current study corroborate prior evidence suggesting that women diagnosed with endometriosis exhibit elevated levels of depression and anxiety in comparison to their healthy peers (e.g. Maulenkul et al., 2024). Specifically, our findings indicate a potential association between PD and concerns related to body image and heightened self-criticism. A key finding is the identification of disparities among women from diverse cultural backgrounds, emphasizing the role of cultural factors in managing endometriosis. This study highlights the importance of addressing self-criticism, including elements like stigma, in the link between body image and PD, suggesting that self-criticism may be an effective target for body image interventions. It could be speculated that in collectivist societies, interventions might be more effective if they focus on community and relational self-worth, rather than solely on individual self-compassion. Such interventions could empower women to navigate the unique challenges of endometriosis while affirming their self-worth (Gilbert, 2002; Gilbert & Procter, 2006).

## Author contributions

S.G., S.L. and R.A conceptualized and designed the study, and supervised the students involved in the collection of the data. S.L. analyzed the data. S.G., S.L. and R.A interpreted the data, and the first version of the manuscript was written by S.G. S.L and R.A rewrote and approved the final version. All authors have read and agreed to the final version of the manuscript.

## Data Availability

The data that support the findings of this study are available on request from the corresponding author (S.G). The data are not publicly available due to restrictions, e.g. their containing information that could compromise the privacy of research participants.
